# HPV presence precedes abnormal cytology in women developing cervical cancer and signals false negative smears

**DOI:** 10.1054/bjoc.2001.1926

**Published:** 2001-08

**Authors:** G D Zielinski, P J F Snijders, L Rozendaal, F J Voorhorst, H C van der Linden, A P Runsink, F A de Schipper, C J L M Meijer

**Affiliations:** Department of Pathology; Epidemiology and Biostatistics, University Hospital Vrije Universiteit, Amsterdam, MB, 1007, The Netherlands; Department of Obstetrics and Gynaecology, Hospital Walcheren, Vlissingen, DD, 4380, The Netherlands; Department of Pathology, District Laboratory Zeeland

**Keywords:** HPV, cervical cancer, archival smears: case–control study, normal cytology, false negative cytology

## Abstract

In a retrospective case–control study, we investigated high-risk HPV DNA presence by general primer GP5+/6+ PCR in the last normal cervical smear in the patient archives (i.e. baseline smear) of 57 women who later developed cervical cancer. Also, normal cervical smears of 114 age-matched control women were analysed. High-risk HPV DNA was detected in 37 of the 57 (65%) baseline smears of the case women, and 7 (6%) of 114 smears of the control women (OR 28, 95% Cl 11–72). The HPV positive subsequent smears and cervical cancer biopsies of the case women contained the same HPV type as was detected in the baseline smear. After cytological revision, the baseline smears of 48 case women (84%) were reclassified as abnormal, 33 (69%) of which scored high-risk HPV DNA positive. Ultimately, an undisputable normal baseline smear was found in only 10 case women. In 7 (70%) of them this smear was HPV positive, whereas only 7 (7%) of 104 revised, undisputable normal smears of control women were high-risk HPV positive (OR 32, 95% Cl 6.8–153). The results showed that (1) high-risk HPV presence precedes abnormal cytology in women who develop cervical cancer, and (2) high-risk HPV testing signals false-negative smears of women at risk of cervical cancer. © 2001 Cancer Research Campaign http://www.bjcancer.com


*British Journal of Cancer* (2001) **85**(3), 398–404
© 2001 Cancer Research Campaign
doi: 10.1054/ bjoc.2001.1926, available online at http://www.idealibrary.com on

### HPV presence precedes abnormal cytology in women developing cervical cancer and signals false negative smears

**GD Zielinski[1], PJF Snijders[1], L Rozendaal[1], FJ Voorhorst[2], HC van der Linden[1], AP Runsink[4], FA de Schipper[3] and CJLM Meijer[1]**

[1]Department of Pathology, [2]Epidemiology and Biostatistics, University Hospital Vrije Universiteit, 1007 MB Amsterdam, The Netherlands; [3]Department of Obstetrics and Gynaecology, Hospital Walcheren, 4380 DD Vlissingen, The Netherlands; [4]Department of Pathology, District Laboratory Zeeland

**Summary** In a retrospective case–control study, we investigated high-risk HPV DNA presence by general primer GP5+/6+ PCR in the last normal cervical smear in the patient archives (i.e. baseline smear) of 57 women who later developed cervical cancer. Also, normal cervical smears of 114 age-matched control women were analysed. High-risk HPV DNA was detected in 37 of the 57 (65%) baseline smears of the case women, and 7 (6%) of 114 smears of the control women (OR 28, 95% CI 11–72). The HPV positive subsequent smears and cervical cancer biopsies of the case women contained the same HPV type as was detected in the baseline smear. After cytological revision, the baseline smears of 48 case women (84%) were reclassified as abnormal, 33 (69%) of which scored high-risk HPV DNA positive. Ultimately, an undisputable normal baseline smear was found in only 10 case women. In 7 (70%) of them this smear was HPV positive, whereas only 7 (7%) of 104 revised, undisputable normal smears of control women were high-risk HPV positive (OR 32, 95% CI 6.8–153). The results showed that (1) high-risk HPV presence precedes abnormal cytology in women who develop cervical cancer, and (2) high-risk HPV testing signals false-negative smears of women at risk of cervical cancer. © 2001 Cancer Research Campaign http://www.bjcancer.com

**Keywords**: HPV; cervical cancer; archival smears: case–control study; normal cytology; false negative cytology

Various molecular and epidemiological data support the notion that persistent infection with high-risk human papillomavirus (HPV) is causally related to the development of cervical cancer and its precursor lesions (Bosch et al, 1995; IARC, 1995; Ho et al, 1998; Nobbenhuis et al, 1999; Walboomers et al, 1999). Further evidence for this well-established link could come from studies showing that infection with high-risk HPV precedes development of cervical premalignant disease in women with cervical cancer. Most follow-up studies performed so far concerning high-risk HPV in women with normal cytology have used the development of precursor lesions of cervical cancer as a surrogate end-point (Koutsky et al, 1992; Rozendaal et al, 1996; Hopman et al, 2000; Josefsson et al, 2000; Ylitalo et al, 2000a,b). To our knowledge only 2 retrospective studies have addressed the issue of high-risk HPV in normal archival smears of women with cervical cancer (Walboomers et al, 1995; Wallin et al, 1999). However, upon revision it appeared that up to 90% of these ‘normal’ archival smears were false-negative ones.

Here, we present the results of a retrospective case–control study using archival smears of women who developed cervical cancer that were defined in 2 ways: (1) the last smear in the patient history that was classified as normal according to the original pathological report, and (2) the last smear in the patient history that was classified as undisputable normal on the basis of both the original and a revised cytological assessment. In addition to these smears and the smears of age-matched control women, all subsequent smears of case women and corresponding cancer biopsies were subjected to high-risk HPV testing by GP5+/6+ PCR-EIA (Jacobs et al, 1997).

The major aim of this study was 2-fold: first, to test the potential value of high-risk HPV testing to signal false-negative cervical smears in women who develop cervical cancer, and second, to find out whether high-risk HPV DNA is present in undisputable normal cervical smears preceding abnormal cytology and whether the respective HPV type persists until cervical cancer.

### MATERIAL AND METHODS

### Patient selection and study design

A population-based cervical cancer screening programme started in 1976 in Zeeland, a district in the Netherlands for which women between the ages of 35 and 54 years were invited. All smears were collected and classified in ‘Streeklaboratorium Zeeland’, the only laboratory of pathology in that area. In this area we started a case–control study. Women were selected with the aid of the laboratory’s manual registry and the national registration system of Pathology in the Netherlands (PALGA), which was introduced in 1986. A total of 187 women were registered to have developed cervical cancer in the period from 1981 to 1998. Of these women, 162 had squamous cell carcinoma and 25 had adenocarcinoma.

Eligible for this study were women with squamous cell carcinoma without a history of cervical disease of whom the first archival smear was registered as normal. Of the 162 women 62

*Received 26 January 2001*
*Revised 25 April 2001*
*Accepted 30 April 2001*

*Correspondence to:* CJLM Meijer had a first smear classified as normal. 5 women were excluded for the following reasons: Absence of archival smears ($n$ = 1), absence of cervical cancer biopsy ($n$ = 1), and inadequacy of available archival smears (taken in 1980, 1984 and 1990, respectively) for DNA detection by PCR ($n$ = 3).

Consequently, a total of 57 case women ultimately took part in this study. 31 (54%) of them had their first smear taken within the screening programme in the period from 1976 to 1996. The remaining 26 women had their first smear taken for medical reasons and opportunistic screening. The median age at the time of diagnosis of cervical cancer was 49 years (range 28–83). Of the 57 women 1 to 8 archival cervical smears were found per case, resulting in a total of 202 smears. According to the original pathological report 139 of the 202 (69%) smears were classified as cytomorphologically normal, of which the large majority was read before 1988. 5 smears were originally classified as inadequate and only 58 of the smears taken closer to diagnosis of cervical cancer were classified as abnormal (mild dyskaryosis or worse). Of the latter, 31 (53%) smears were read after 1990. 2 case–control studies were performed:

(1) For the initial case–control study the last smear in the patient history that was classified as normal according to the original pathological report was selected for HPV analysis, and this smear is indicated as 'baseline smear'. The median age of the case women at the time of the baseline smear was 41 years (range 20–73).

(2) The second case–control study involved the last smear in the patient history that was classified as undisputable normal on the basis of both the original and a revised cytological assessment. This smear is indicated as 'revised baseline smear'.

In addition, for HPV analysis all available smears subsequent to the normal smear as well as corresponding formalin-fixed cancer biopsies were used as well. A flow chart of the whole study design is given in Figure 1.

### Selection of control women

For the initial case–control study involving baseline smears 2 control women were selected per case women from women who participated in the screening programme in the same area in Zeeland (Figure 1). The control women ($n$ = 114) did not develop cervical cancer within the same time interval as the case women. If possible, a normal smear was selected of the control women when they had the same age as the case women at the time their baseline smear was taken. If controls of the same age were not available, a control was selected with an age as close as possible to the age of the case woman. Optimal selection of controls was limited by the laboratory's manual registry, the start of cervical cancer screening not earlier than 1976, and the law enforcement to keep cervical smears for 10 years. Therefore, we were unable to use cervical smears taken after 1986 of women who did not develop cervical cancer. The median age of the control women was 43 years (range 34–54). This was not significantly different from the age of the case women (41 years with a range of 20 to 73 years).

For the second case–control study involving revised baseline smears, the controls were used of whom the smear remained normal after revision of cytology ($n$ = 104).

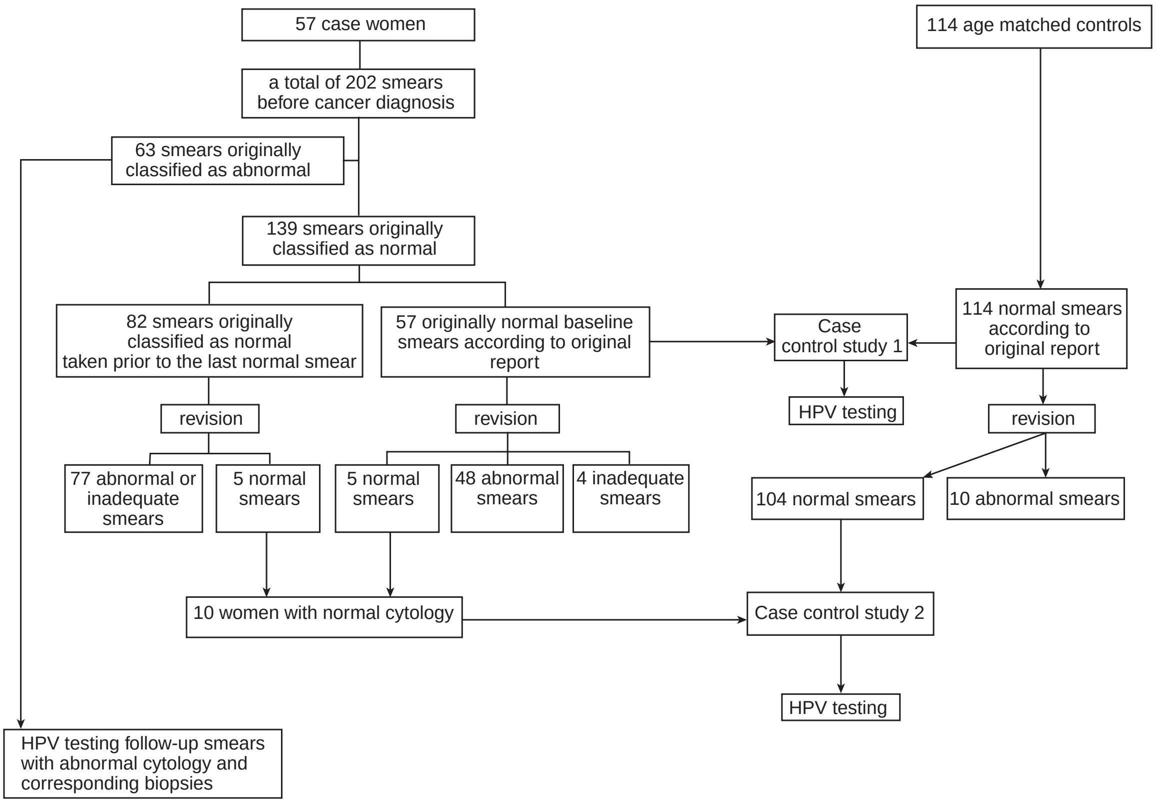

**Figure 1** Flow chart of cases/controls and study design

### Revision of cytology and histology and quality of the smears

All originally normal smears were revised blindly, by mixing smears of cases and controls randomly, according to the KOPAC classification as described before (Vooijs, 1987). Briefly: Pap 0 is inadequate material for review, Pap 1 is normal cytology, Pap 2 is very mild dyskaryosis, Pap 3a1 is mild dyskaryosis, Pap 3a2 is moderate dyskaryosis, Pap 3b is severe dyskaryosis, Pap 4 is suspect for carcinoma in situ and Pap 5 is suspect for invasive cancer. Since the detection of HPV DNA may depend on the number of epithelial cells, this number was crudely estimated by microscopical assessment of the proportion of epithelial cells in several representative microscopic fields using a point-counting raster and multiplying this value with the number of cells in the smear. The latter was estimated on the basis of a comparison with a more or less ideal smear containing about 2 million cells.

The cervical cancer biopsy specimens were histopathologically reviewed to confirm the diagnosis.

### DNA extraction and HPV detection by PCR

Archival smears were processed using the High Pure PCR Template Preparation (HPPTP) extraction assay, as described previously (Jacobs et al, 2000). HPV detection in the smears of cases and controls was blinded by mixing them at random.

Of the formalin-fixed biopsies five to ten 5 μm thick sections of the cancer biopsies were cut, according to the sandwich-method. This method allows a histopathological assessment to ascertain that tissue sections processed for HPV detection contain cancer cells (Walboomers et al, 1997). Further processing was as described before (Jacobs et al, 1997).

A β-globin PCR generating a 209 base pair product was performed on all smears and cancer biopsies, to determine DNA integrity of the samples (De Roda Husman et al, 1994). If samples of the cancer biopsies were β-globin negative, the HPPTP extraction assay was used to clear the DNA from possible PCR inhibitors. Only samples that were ß-globin PCR positive were subjected to high risk HPV analysis. High-risk HPV detection was performed by GP5+/6+ PCR EIA as described before (Jacobs et al, 1997, 2000; Walboomers et al, 1997).

High-risk HPV genotypes were first determined as a group using a cocktail of HPV type-specific oligoprobes, followed by individual typing for 14 high-risk HPV types (16, 18, 31, 33, 35, 39, 45, 51, 52, 56, 58, 59, 66 and 68). The cut-off for HPV positivity was set to 3 times the average optical density of PCR negative controls (Jacobs et al, 1997).

For HPV detection in the corresponding biopsy specimen the GP5+/6+ PCR EIA was supplemented with E7 region specific PCR assays as described before, since in cancer biopsies the L1 based GP5+/6+ assay may fail to reveal the presence of HPV DNA due to possible integration events of the viral genome, which occurs in about 3% of the cases (Walboomers et al, 1999).

### Statistical analysis

The odds ratios (OR) and their confidence intervals were estimated by logistic regression analysis. Differences between the investigated continuous variables were tested using the Mann–Whitney U test or Kruskal–Wallis test and between categorical variables using the Fisher test. All reported *P* values were 2-sided. To estimate the time-interval of infection with high-risk HPV to diagnosis of cervical cancer, the smears taken 0.5 years or less before diagnosis of cervical cancer, were excluded.

### RESULTS

### HPV detection in baseline smears of cases and normal smears of controls

High-risk HPV DNA was detected in 37 of the 57 (65%) baseline smears of the case women (Table 1). HPV 16, 18 and HPV 31 DNA were present in 29, 3 and 3 baseline smears, respectively, and HPV 33 and HPV 45 were each present in 1 of the baseline smears. In contrast, high-risk HPV DNA was detectable in only 7 of the 114 (6%) smears analysed of the control women. In these smears HPV 16, 18 and HPV 31 were each found twice and HPV 45 once. The prevalence of high-risk HPV DNA was significantly less in the control group than in the case group ($P < 0.001$, Fisher test). The crude odds ratio for high-risk HPV DNA in smears classified as normal according to the original pathological report to predict development of cervical squamous cell carcinoma was 28 (95% CI 11–72; Table 1).

### HPV detection in subsequent smears and biopsies of case women

Of the 57 case women 52 ß-globin PCR positive abnormal smears were available that were taken after the baseline smears but prior to diagnosis of cervical cancer. 38 (73%) of them were high-risk HPV positive. In 52 of the 57 (91%) women the corresponding cervical carcinoma biopsies, revealed high-risk HPV positivity. A total of 41 (72%) women had high-risk HPV in the baseline and/or subsequent smears and corresponding biopsies (Table 2). In all HPV positive cases, the same HPV type was present in the baseline smears, subsequent smears and corresponding biopsies that scored positive by high-risk HPV PCR. Based on HPV status in smears and cancer biopsy, women could be placed into 4 groups. The first group ($n = 33$) consisted of women with both baseline, follow-up smears (when available) and corresponding cervical cancer biopsy consistently HPV positive. The second group ($n = 8$)

**Table 1** High risk HPV in baseline and control smears, age of women and storage time

| | Number of case women | Number of control women | Odds ratio |
|---|---|---|---|
| HPV+ (%) | 37 (65%) | 7 (6%) | 28 (95% CI 11–72)[a] |
| HPV– (%) | 20 (36%) | 107 (94%) | 1 |
| Median age in years (range) | 41 (20–73) | 43 (34–54) | |
| Median storage time in years (range) | 16.5 (0.9–35.0) | 20.9 (14.5–23.1) | |

[a]The crude odds ratio for high-risk HPV DNA in baseline smears classified as normal according to the original pathological report to predict development of carcinoma.

 © 2001 Cancer Research Campaign

**Table 2** Results of HPV detection in baseline and follow-up smears of 57 case women

| Number of smears of case women (number of women) | Number of case women | | | | |
|---|---|---|---|---|---|
| | cxca[a] HPV+ | | | cxca HPV– | Total number of smears (number of HPV + smears) |
| | All smears HPV+ | Smears HPV+ and HPV– | All smears HPV– | All smears HPV– | |
| 1 (28) | 16 | | 9 | 3 | 28 (16) |
| 2 (17) | 9 | 5 | 2 | 1 | 34 (23) |
| 3 (7) | 4 | 2 | | 1 | 21 (14) |
| 4 (2) | 2 | | | | 8 (8) |
| 5 (1) | | 1 | | | 5 (2) |
| 6 (1) | 1 | | | | 6 (6) |
| 7 (1) | 1 | | | | 7 (7) |
| (57) | 33 (58%) | 8 (14%) | 11 (19%) | 5 (9%) | 109 (76 HPV+, 70%) |

[a]cxca = cervical carcinoma.

**Table 3** HPV detection in baseline and follow-up smears of case women stratified for years before diagnosis of cervical cancer

| Years before diagnosis of cervical cancer | HPV– smears | HPV+ smears | Total number of smears |
|---|---|---|---|
| 0–3 | 12 (24%) | 38 (76%) | 50 |
| 4–6 | 7 (37%) | 12 (63%) | 19 |
| 7–20 | 14 (34%) | 27 (66%) | 41 |
| Total | 33 (30%) | 76 (70%) | 109 |

**Table 4** Revision of baseline smears in relation to HPV status and number of epithelial cells

| Revision according to KOPAC-B | Number of case women | %HPV+ (n HPV+) | Number of epithelial cells (range) $\times 10^4$ | |
|---|---|---|---|---|
| | | | HPV+ | HPV–[a] |
| Inadequate (Pap 0) | 4 (7%) | 0% (0) | | 5.9 (0.9–36) |
| Normal (Pap 1) | 5 (9%) | 80% (4) | 24.9 (2.5–115.2) | 5.4 |
| Very mild dyskaryosis (Pap 2) | 19 (33%) | 58% (11) | 20.0 (4.5–96) | 20.5 (20–90) |
| Mild dyskaryosis (Pap 3a1) | 5 (9%) | 20% (1) | 57.8 | 35.2 (12.1–78.4) |
| Moderate dyskaryosis (Pap 3a2) | 10 (17%) | 80% (8) | 75.8 (15.0–99.2) | 10.5 (8.4–12.6) |
| Severe dyskaryosis (Pap 3b) | 12 (21%) | 92% (11) | 38 (6.3–160) | 9.6 |
| Suspect for carcinoma in situ (Pap 4) | 1 (2%) | 100% (1) | 49 | |
| Suspect for carcinoma (Pap 5) | 1 (2%) | 100% (1) | 60 | |
| Total | 57 | 65% (37) | 40 (2.5–160) | 12.4 (0.9–90) |

[a]The number of epithelial cells in high-risk HPV negative smears was significantly lower than in high-risk HPV positive cervical smears (KW $P = 0.018$).

included women with follow-up smears in which the presence of HPV fluctuated in the smears, but of whom the cancer biopsy was HPV positive. 5 of these women had 2 smears of whom 3 had a first smear negative for high-risk HPV and a second smear and corresponding cancer biopsy positive for high-risk HPV. The other 2 case women had high-risk HPV detectable in the first smear and corresponding cancer biopsy, but not in the second smear. 2 other case women of this group had a first smear and corresponding cancer biopsy positive for high-risk HPV DNA, but their second and third smear were negative. The last case woman within this group had a first and third smear, and corresponding cancer biopsy positive for high-risk HPV DNA, whereas the second, fourth and fifth smear were high-risk HPV negative. The third group ($n = 11$) included women with all smears being HPV negative, but a cancer biopsy that was positive for high-risk HPV. The fourth group ($n = 5$) comprised women of whom both smears and cancer biopsies were negative for high-risk HPV. Although the prevalence of high-risk HPV in the follow-up smears tended to be highest in the smears taken closest to diagnosis of cervical cancer (76% HPV positivity in smears taken less then 3 years prior to cancer diagnosis) the difference with smears taken at an earlier time point was not significant (KW $P = 0.316$, Table 3).

### Abnormal cytology after revision of the baseline smears in relation to presence of high-risk HPV

All baseline smears and smears of control women were revised according to the KOPAC-B classification currently used in the Netherlands (Table 4). The baseline smears of 48 case women (84%) were reclassified as abnormal (Pap 2 or worse), 4 (7%) were inadequate for cytology and only 5 (9%) remained normal. Of the 48 revised abnormal baseline smears of the case women 33 (69%) revealed the presence of high-risk HPV. The median time to diagnosis of cervical cancer was 8.3 years (0.5–19.2 years) for the revised abnormal baseline smears. Of the 24 smears reclassified as moderate dyskaryosis or worse 21 (88%) were HPV positive. The 3 smears in this group in which no HPV DNA could be detected included 2 of the 10 Pap 3a2 (moderate dyskaryosis) and 1 of the 12 (8%) Pap 3b (severe dyskaryosis) smears. In contrast, only 10 of the 114 (9%) smears of the control women were revised as abnormal; 8

*© 2001 Cancer Research Campaign* 

**Table 5** High risk HPV in revised baseline and control smears

| | Number of case women (%) | Number of control women (%) | Odds ratio |
|---|---|---|---|
| HPV+ | 7 (70%) | 7 (7%) | 32 (95% Cl 6.8–153)[a] |
| HPV– | 3 (30%) | 97 (93%) | 1 |
| Total | 10 | 104 | |

[a]The crude odds ratio for high-risk HPV DNA in smears revised as normal according to the KOPAC-B classification to predict development of carcinoma.

**Table 6** Characteristics of case women with revised baseline smears

| Case | HPV type in revised baseline smear | Years before cervical cancer | HPV positive/total no.of smears | HPV type in cancer biopsy |
|---|---|---|---|---|
| 8 | 31 | 5.8 | 2/2 | 31 |
| 26 | 16 | 9.6 | 4/4 | 16 |
| 28 | 16 | 10.8 | 3/3 | 16 |
| 45 | – | 15.2 | 0/2 | 18 |
| 67 | 16 | 17.4 | 1/1 | 16 |
| 72[a] | 18 | 10.8 | 6/9 | 18 |
| 81 | – | 8.6 | 1/5 | 18 |
| 83[b] | 16 | 9.7 | 3/4 | 16 |
| 87 | – | 10.7 | 0/2 | 16 |
| 88[c] | 16 | 10.3 | 4/4 | 16 |
| Total | | | 24/36 (64%) | 10/10 |

[a]Case 72 had 1 cytomorphologically normal smear that was HPV negative 14.8 years before diagnosis of cervical cancer, with a storage time of 22.5 years. [b]Case 83 had 2 cytomorphologically normal smears of which one was HPV positive 11.7 years before diagnosis of cervical cancer, with a storage time of 15.3 years and the other taken 14.3 years before diagnosis of cervical cancer, with a storage time of 18.0 years was HPV negative. [c]Case 88 had 1 cytomorphologically normal smear that was HPV positive 13.8 years before diagnosis of cervical cancer, with a storage time of 14.3 years.

of them were reclassified as very mild dyskaryosis (Pap 2), 1 as mild dyskaryosis (Pap 3a1) and 1 as severe dyskaryosis (Pap 3b).

### HPV presence in revised abnormal smears in relation to the storage time and the number of epithelial cells

In order to find out what phenomenon may underlie the non-detection of HPV in the 15 HPV negative, revised abnormal baseline smears the storage time and number of epithelial cells were analysed in relation to HPV detection (Table 4). The number of epithelial cells was significantly lower in the high-risk HPV-negative (median $1.2 \times 10^5$, range $9.0 \times 10^3$–$9.0 \times 10^5$) compared to high-risk HPV-positive baseline smears (median $4.0 \times 10^5$, range $3 \times 10^4$–$1.6 \times 10^6$; KW $P = 0.018$, Table 4). No significant differences in storage time of HPV negative (20.5 years, range 7.1–24.7 years) versus HPV positive (19.6 years, range 9.4–35 years) smears was evident ($P = 0.54$. Mann–Whithey). Also the median duration of taking the abnormal smears to diagnosis of cervical cancer did not differ significantly between HPV negative (9.0 years, range: 0.5–19.0 years) and HPV positive (8.0, range 0.5–19.2 years) abnormal baseline smears ($P = 0.17$, Mann–Whitney).

### HPV detection in undisputable normal baseline smears after revision

High-risk HPV was present in the baseline smear of 4 (80%) of the 5 case women of whom this smear remained normal after revision. 5 other case women had a smear that was revised as normal, taken prior to the baseline smear previously defined. High-risk HPV DNA was detected in 3 of these smears. Therefore, the total number of case women that ultimately remained with an undisputable normal baseline smear after revision was 10, 7 (70%) of which revealed high-risk HPV positivity. 7 (7%) of 104 revised normal smears of the controls were HPV positive. The crude odds ratio for high-risk HPV DNA detection in revised baseline smears to predict development of carcinoma was 32 (95% Cl 6.8–153; Table 5). The median duration to diagnosis of cervical cancer for the HPV positive revised baseline smears was 10.7 years (range 9.6–17.4). This was not markedly different for revised baseline smears that were HPV negative (10.7 years, range 8.6–15.2 years). However, the high-risk HPV-negative baseline smears revealed a lower number of epithelial cells than the high-risk HPV positive smears (median $6.4 \times 10^4$, range $5.4 \times 10^4$–$12.8 \times 10^4$ and median $16.2 \times 10^4$, range $2.6 \times 10^4$–$1.2 \times 10^5$, respectively).

High-risk HPV was furthermore present in 1 or more of the follow-up smears of 8 women (Table 6). Of all women the corresponding cervical cancer biopsy was HPV positive. Typing revealed HPV 16 in 6, HPV 18 in 3 and HPV 31 in 1 of the carcinomas. The same HPV types were present in corresponding HPV positive smears.

Based on HPV status in smears and cancer biopsy, women could be placed into 3 groups. The first group ($n = 5$) consisted of women with both baseline, follow-up smears (when available) and corresponding cervical cancer biopsy consistently HPV positive. The second group ($n = 3$) comprised women with follow-up smears in which the presence of HPV fluctuated and of whom the cancer biopsy was HPV positive. One of these case women had a high-risk HPV negative normal smear and a subsequent abnormal smear that was HPV positive 5.7 years before diagnosis of cervical cancer (Table 6, case women 81). The third group ($n = 2$) comprised women with HPV negative smears but a cancer biopsy that was positive for high-risk HPV (Table 6, case woman 45 and case woman 87).

 © 2001 Cancer Research Campaign

Of 3 case women normal smears were available that preceded the HPV positive revised baseline smear. In one of them that smear was HPV positive 13.8 years before diagnosis of cervical cancer (Table 6, case woman 88). Another had 2 preceding normal smears, one of which was HPV positive 11.7 years before diagnosis of cervical cancer and another taken 14.3 years before cancer diagnosis was HPV negative (Table 6, case woman 83). A third woman had an earlier HPV negative normal smear taken 14.8 years before cancer diagnosis (Table 6, case woman 72).

### DISCUSSION

This retrospective population based case–control study revealed that high-risk HPV testing signals 69% of the false negative archival smears (33 of 48 revised abnormal baseline smears were HPV positive) of women who developed cervical cancer. High-risk HPV DNA was also detectable in undisputable normal smears preceding cervical cancer in 7 of the 10 women analysed. The same HPV type was detected in revised baseline smears, subsequent smears and corresponding biopsies that were HPV positive. This does not only provide further evidence that high-risk HPV precedes the development of premalignant disease leading to cervical cancer, but also supports the notion that viral persistence is essential in this process. Furthermore, we found that high-risk HPV positivity in revised normal cervical smears was strongly associated with cervical cancer development (OR 32, 95% CI 6.8–153). However, it should be kept in mind that this figure may be overestimated since a subset of controls used in this analyses was of older age than the corresponding cases. It is well known that the HPV prevalence is higher among younger women (Melkert et al, 1993).

The clinical implications of our findings are 2-fold: First, HPV testing may serve as a quality control for cytology. This is supported by the recent finding that revision of HPV-positive normal smears of a screening population yielded abnormal smears in 5% to 7% of the cases (Meijer et al, 1997). Second, HPV testing allows the recognition of women at risk prior to the manifestation of abnormal cytology. In the 7 women with HPV positive undisputable normal baseline smears, the median duration until the diagnosis of cervical cancer was 10.7 years (8.6–15), which was at least 2 years longer than that of the manifestation of abnormal cytology. This finding suggests that the current time-interval to the next screening round (which is 5 years in the Netherlands) for women with HPV-negative normal cytology in cervical cancer screening programmes could be prolonged. However, the number of women studied for assessment of the time interval between the normal smear and cervical cancer should be increased before an accurate statement about this time interval can be given.

Similar to other studies, a considerable proportion of baseline smears of case women that were originally classified as normal, were reclassified as abnormal after revision (Walboomers et al, 1995; Wallin et al, 1999). In contrast, the far majority (95%) of smears of the control women remained normal after revision. Reasons for previous false-negative smears, of which the far majority was read before 1988 when the KOPAC classification was introduced, may include technical failures like screening fatigue and very few dyskaryotic cells present in the smears. In addition, the imperfection of former cytology, i.e. the ignorance of tissue fragments, may, in part, be responsible. Since former training programmes concentrated almost exclusively on cytology of dissociated cells, no attention was paid to the identification of tissue fragments (Robertson and Woodend, 1993). It is noteworthy that we more than incidently observed tissue fragments in the smears that were revised as abnormal. In conclusion, it is likely that the use of current criteria of cytology would have yielded fewer false negative smears among the case women in this study.

Although the great majority of false-negative baseline smears were positive for high-risk HPV still 15 of them scored HPV negative. However, it is of note that we found that the number of epithelial cells was significantly lower in the HPV-negative compared to the positive baseline smears. Moreover, the extraction method we used, yields at maximum amplifiable DNA of 40% of the total DNA content of a given smear (Jacobs et al, 2000). Wallin et al (1999) attributed underestimation of HPV positivity in archival smears of cancer patients to storage time, and the fact that HPV detection was performed on archival smears instead of fresh samples (Cuzick et al, 2000). Taken together, it is likely that factors like low number of epithelial cells, relatively low yields of extracted DNA and reduced DNA quality of stored smears alone or in combination, may contribute to an underestimation of HPV presence in archival smears. Therefore, the analysis of fresh rather than archival smears probably would have signalled even more cytomorphologically false negative cases (Cuzick et al, 2000).

In our previous retrospective study on a smaller series of archival smears of case women from another area in the Netherlands high-risk HPV DNA was detected in a higher (i.e. 89%) percentage of baseline smears (Walboomers et al, 1995). However, after revision all adequate smears in that study were reclassified as severe dyskaryosis or worse. In this study, the baseline smears that were revised as severe dysplasia or worse also revealed a higher HPV prevalence (Table 4). In contrast, the percentage of HPV positive baseline smears was markedly lower (i.e. about 30%) in a study of Wallin et al (1999). Here, also the number of smears that were reclassified as dyskaryotic after revision was lower. Although technical differences, such as the use of different DNA isolation procedures may partially underlie the different prevalence rates, these findings are in favour of the idea that differences in HPV prevalence in smears preceding cervical cancer can also be due to differences in the proportion of dyskaryotic smears (Walboomers et al, 1995).

Another finding was that the HPV prevalence in the cervical carcinomas (91%) in this study was lower than previously reported for a worldwide study, in which 4 different HPV primer sets were used (Bosch et al, 1995; Walboomers et al, 1999). However, it should be kept in mind that our study is based on the analysis of samples that were not optimal, i.e. archival, formalin-fixed tissue samples that had been stored for quite some time.

In conclusion, this study unambiguously demonstrated the presence of the same HPV type in undisputable normal and subsequent abnormal smears until diagnosis of cervical cancer, and thus showed that high-risk HPV detection precedes the development of abnormal cytology. Since the duration to cervical cancer is about 10 years for an undisputable normal smear these data suggest that the time interval between 2 screening rounds can be increased from 5 years (as it is now in The Netherlands) to 8–10 years. Furthermore, since in this study high-risk HPV testing signalled 69% of the abnormal smears originally misclassified as normal, of the women who developed cervical cancer, we suggest to rescreen all high-risk HPV-positive cytomorphologically normal smears. In that case, HPV testing provides a safety net for false negative smears of women who are at risk of cervical cancer.

*© 2001 Cancer Research Campaign*

### ACKNOWLEDGEMENTS

This work is dedicated to the memory of Jan MM Walboomers who passed away on the 2 February 2000. We are indebted to Dr Marcel Jacobs and Mr René Pol for technical assistance, and to F Peters for assistance of sample collection. This work was supported by grant number 28-2831 of the Dutch Prevention Fund/ZorgOnderzoekNederland (ZON).

### REFERENCES

Bosch FX, Manos MM, Muñoz N, Sherman M, Jansen AM, Peto J, Schiffman MH, Moreno V, Kurman R and Shah KV (1995) Prevalence of human papillomavirus in cervical cancer: a worldwide perspective. *J Natl Cancer Inst* **87**: 796–802

Cuzick J, Terry G, Ho L, Monaghan J, Lopes A, Clarkson P and Duncan I (2000) Association between high-risk HPV types, HLA DRB1* and DQB1* alleles and cervical cancer in British women. *Br J Cancer* **82**: 1348–1352

De Roda Husman A-M, Walboomers JMM, Meijer CJLM, Risse EKJ, Schipper MEI, Helmerhorst ThJM, Bleker OP, Delius H, van den Brule AJC and Snijders PJF (1994) Analysis of cytomorphologically abnormal cervical scrapes for the presence of 27 mucosotropic human papillomavirus genotypes, using polymerase chain reaction. *Int J Cancer* **56**: 802–806

Ho GYF, Bierman R, Beardsley L, Chang CJ and Burk RD (1998) Natural history of cervicovaginal papillomavirus infection in young women. *N Engl J Med* **338**: 423–428

Hopman EH, Rozendaal L, Voorhorst FJ, Walboomers JMM, Kenemans P and Helmerhorst ThJM (2000) High risk HPV in women with normal cervical cytology prior to the development of abnormal cytology and colposcopy. *BJ Obstet Gynaecol* **107**: 600–604

IARC (1995) Monographs on the evaluation of carcinogenic risks to humans. Human papillomaviruses, Vol. 64, Human papillomaviruses. Lyon: International Agency for Research on Cancer

Jacobs MV, Snijders PJF, van den Brule AJC, Helmerhorst ThJM, Meijer CJLM and Walboomers JMM (1997) A general primer GP5+/GP6+ mediated PCR-enzyme immunoassay method for rapid detection of 14 high-risk and 6 low-risk human papillomavirus genotypes in cervical scrapings. *J Clin Microbiol* **35**: 791–795

Jacobs MV, Zielinski GD, Meijer CJLM, Pol RP, Voorhorst FJ, de Schipper FA, Runsink AP, Snijders PJF and Walboomers JMM (2000) A simplified and reliable HPV testing of archival Papaniculaou-stained archival smears: application to cervical smears from cancer patients starting with cytologically normal smears. *Br J Cancer* **82**: 1421–1426

Josefsson AM, Magnusson PKE, Ylitalo N, Sorensen P, Qwarforth-Tubbin P, Kragh Andersen P, Melbeye M, Adami HO and Gyllensten UB (2000) Viral load of human papilloma virus 16 as a determinant for development of cervical carcinoma in situ: a nested case-control study. *Lancet* **355**: 2189–2193

Koutsky LA, Holmes KK, Critchlow CW, Stevens CE, Paavonen J, Beckmann AM, DeRouen TA, Galloway DA, Vernon D and Kiviat NB (1992) A cohort study of the risk of cervical intraepithelial neoplasia grade 2 or 3 in relation to papillomavirus infection. *N Engl J Med* **327**: 1272–1278

Meijer CJLM, Rozendaal L, Van der Linden JC, Helmerhorst ThJM, Voorhorst FJ and Walboomers JMM (1997) Human papillomavirus testing for primary cervical cancer screening. In: Franco E, Monsonego J. New developments in cervical cancer screening and prevention. Oxford; *Blackwell Science* 338–347

Melkert PWJ, Hopman E, Van den Brule AJC, Risse EKJ, Van Diest PJV, Bleker OP, Helmerhorst T, Schipper MEI, Meijer CJLM and Walboomers JMM (1993) Prevalence of HPV in cytomorphologically normal cervical smears, as determined by the polymerase chain reaction, is age dependent. *Int J Cancer* **53**: 919–923

Nobbenhuis MAE, Walboomers JMM, Helmerhorst ThJM, Rozendaal L, Remmink AJ, Risse EK, Van der Linden JC, Voorhorst FJ, Kenemans P and Meijer CJLM (1999) Relation of human papillomavirus status to cervical lesions and consequences for cervical-cancer screening: a prospective study. *Lancet* **354**: 20–25

Robertson JH and Woodend B (1993) Negative cytology preceding cervical cancer: causes and prevention. *J Clin Pathol* **46**: 700–702

Rozendaal L, Walboomers JMM, Van der Linden JC, Voorhorst FJ, Kenemans P, Helmerhorst ThJM, van Ballegooijen M and Meijer CJLM (1996) PCR-based high-risk HPV test in cervical cancer screening gives objective risk assessment of women with cytomorphologically normal cervical smears. *Int J Cancer* **68**: 766–769

Vooijs GP (1987) De advisering bij afwijkende bevindingen van cytologisch onderzoek van de cervix uteri. *Ned Tijdschr Geneeskd* **131**: 1662–1663

Walboomers JMM, De Roda Husman A-M, Snijders PJF, Stel HV, Risse EKJ, Helmerhorst ThJM, Voorhorst FJ and Meijer CJLM (1995) Human papillomavirus testing in false negative archival cervical smears: implications for screening for cervical cancer. *J Clin Pathol* **48**: 728–732

Walboomers JMM, Jacobs MV, Van Oostveen JW, Van den Brule AJC, Snijders PJF and Meijer CJLM (1997) Detection of genital HPV infections and possible clinical implication. In: Gross G, Von Krogh G, editors. Human papillomavirus in dermatovenereology. New York: CRC Press, Boca Raton p. 341–364

Walboomers JMM, Jacobs MV, Manos MM, Bosch FX, Kummer JA, Shah KV, Snijders PJF, Peto J, Meijer CJLM and Muñoz N (1999) Human papillomavirus is a necessary cause of invasive cervical cancer worldwide. *J Pathol* **189**: 12–19

Wallin KL, Wiklund F, Angström T, Bergman F, Stendahl U, Wadell G, HAllmans G and Dillner J (1999) Type-specific persistence of human papillomavirus DNA before the development of invasive cervical cancer. *N Engl J Med* **341**: 1633–1638

Ytitalo N, Josefsson A, Melbye M, Sörensen P, Frisch M, Kragh Andersen P, Sparén P, Gustafsson M, Magnusson P, Pontén J, Gyllensten U and Adami H-O (2000a) Prospective study showing long-term infection with human papillomavirus 16 before the development of cervical carcinoma in situ. *Cancer Res* **60**: 6027–6032

Ylitalo N, Sorensen P, Josefsson AM, Magnusson PKE, Kragh Andersen P, Pontén J, Adami HO, Gyllenstenuo and Melbye M (2000b). Consistent high viral load of human papilloma virus 16 and risk of cervical carcinoma in situ: a nested case-control study. *Lancet* **355**: 2194–2198

 © 2001 Cancer Research Campaign
